# Optimized artificial neural network to improve the accuracy of estimated fault impedances and distances for underground distribution system

**DOI:** 10.1371/journal.pone.0227494

**Published:** 2020-01-30

**Authors:** Kanendra Naidu, Mohd Syukri Ali, Ab Halim Abu Bakar, Chia Kwang Tan, Hamzah Arof, Hazlie Mokhlis

**Affiliations:** 1 Electrical Technology Section, Universiti Kuala Lumpur, British Malaysian Institute, Gombak, Selongor Darul Ehsan, Malaysia; 2 Higher Institution Centre of Excellence (HICoE), UM Power Energy Dedicated Advanced Centre (UMPEDAC), Level 4, Wisma R&D, University of Malaya, Jalan Pantai Baharu, Kuala Lumpur, Malaysia; 3 Department of Electrical Engineering, Faculty of Engineering, University of Malaya, Kuala Lumpur, Malaysia; Politechnika Krakowska im Tadeusza Kosciuszki, POLAND

## Abstract

This paper proposes an approach to accurately estimate the impedance value of a high impedance fault (HIF) and the distance from its fault location for a distribution system. Based on the three-phase voltage and current waveforms which are monitored through a single measurement in the network, several features are extracted using discrete wavelet transform (DWT). The extracted features are then fed into the optimized artificial neural network (ANN) to estimate the HIF impedance and its distance. The particle swarm optimization (PSO) technique is employed to optimize the parameters of the ANN to enhance the performance of fault impedance and distance estimations. Based on the simulation results, the proposed method records encouraging results compared to other methods of similar complexity for both HIF impedance values and estimated distances.

## Introduction

Underground distribution systems are widely implemented due to a higher level of security against environmental hazards. However, identifying the HIF location in an underground system is difficult due to the low fault current and non-visibility [1]. Therefore, a fast and accurate HIF fault localization in an underground system is crucial to reduce the risk of damage proliferation, outage time and accelerate the restoration process [2]. Estimating the impedance and location of HIF is a challenging task due to the similarity of the operating current magnitude under normal and faulty conditions. Thus, the low fault current is insufficient to trigger the conventional overcurrent protection relay [3, 4]. Furthermore, it is reported that about 5% to 10% of all the fault events in the distribution system are caused by HIF. However, the actual percentage could be higher since only HIF events ending in bolted faults are recorded [5].

Various fault location methods have been proposed to identify the location of HIFs [6–8]. The literature concludes that the fault location error grows as the fault impedance value and the distance to the fault point increase. Impedance-based methods have been used to locate HIFs eventhough these methods are more suitable for locating low impedance faults (LIF). Besides the impedance-based method, high frequency travelling wave, analytical formulation, fundamental component-based, and knowledge-based methods have also been introduced to tackle the HIF localization problem.

The high frequency travelling wave method is commonly used for the transmission line. In a distribution system, this method is difficult to be implemented due to the complex topology of the network. A distribution system with lateral branches will cause many refractions and reflections from discontinuity points and at the lateral junctions [9]. To overcome this difficulty, a new approach involving signal processing method and artificial intelligence (AI) methods are proposed in [10–16]. In these hybrid methods, the extracted features from the fault generated travelling wave is fed into an AI technique to determine the fault location. Several types of signal processing methods have been proposed to extract the features from the measured voltage and current signals. Wavelet transform (WT) is the most popular tool used in this application as it provides both time and frequency information of the measured signal [17, 18]. On the other hand, empirical mode decomposition (EMD) has been proposed in [19] to extract the transient signal. In [20], the discrete Fourier transform (DFT) is used instead of WT and EMD for the same purpose. Similarly, several AI methods have also been proposed such as artificial neural network (ANN) [21, 22], fuzzy logic system (FLS) [23, 24], support vector regression (SVR) [10, 25–27], core vector regression (CVR) [19] and adaptive neuro-fuzzy inference system (ANFIS) [2, 20]. Some researchers have also utilized more than one AI method as proposed in [23–25]. In [12], the relationship between the path characteristic frequencies and fault location is investigated. Whereas in [14, 28], the frequency spectrum of the voltage waveform is used to estimate the fault distance. Besides that, the analytical formulation method has been utilized in [29–31] to estimate the fault distance. In [31], an estimation based approach using equation derivation is formulated. Whereas in [29], the analytical formulation method based on a forward calculation of zero sequence voltage and current has been proposed. Other than that, the bus impedance matrix of the system is derived in [30] to estimate the distance. All the mentioned techniques have their own strength and weakness in estimating the fault distance accurately. It can be observed that some of them are only applicable to the transmission system or LIF event. There are a few proposed techniques that require complex computation to solve the problem. Regardless of all the weaknesses, all the proposed techniques have contributed a significant finding to solve the fault distance estimation problem.

In this paper, the estimation of fault impedance and distance values due to the occurrence of a single line to ground fault (SLGF) and balanced fault in the underground distribution system is proposed. A single measurement of three-phase voltage and current waveforms are measured at the main substation. The DWT is used to extract the features from the measured voltage and current waveforms. Then, the correlation between the extracted features of voltage and current waveforms is obtained using the cross-product analysis. Subsequently, the ANN is utilized to estimate the fault impedance and distance values. A thorough investigation is carried out to analyze the effect of different types of ANN learning algorithms as well as its ANN parameters such as learning rate (*lr*), momentum constants (*mc*) and the number of neurons in a hidden layer. The novelty of this paper is the implementation of the PSO technique to determine the optimal values of these various ANN parameters in order to evaluate the value of fault impedance and its distance. The determination of these optimal ANN parameters is not considered before, which significantly improves the performance of ANN to obtain a higher level of accuracy compared to conventional ANN.

## Background

The proposed method detects the occurrence of HIF and estimates its impedance and distance values using a combination of DWT, PSO and ANN. As such, a brief description of DWT, PSO and ANN is explained as follows.

### Discrete wavelet transforms

Discrete wavelet transforms (DWT) is a mathematical function that transforms the original signal into time and frequency domain components. This unique ability of DWT to obtain both time and frequency domain information overcomes the limitation of other signal processing techniques such as Fourier Transform [18].

**Fig 1:**
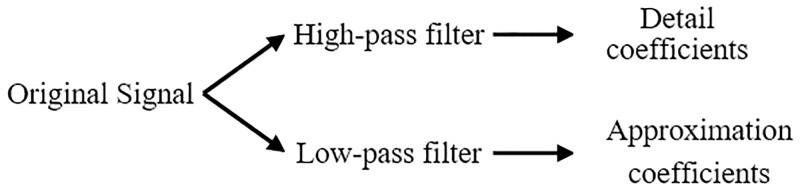
Discrete wavelet transform decomposition process.

Fig 1 shows the basic operation of DWT, which consists of two complementary filters which are high-pass and low-pass filters. Both filters decompose the original signal into high-frequency and low-frequency components. The low-frequency component which is known as approximation coefficients resembles the original signal. Whereas, the high-frequency component which is also known as detail coefficients shows the fast variations in the signal. The high-frequency component, *y*_*high*_ and the low-frequency component, *y*_*low*_ for the original signal, *x(k)* passes through the high-pass filter, *h(2n-k)* and low-pass filter, *l(2n-k)* with downsampling by a value of two (2) are shown by the following equations:

$$y_{high}(n)=\sum_{k=-\infty }^{\infty }x(k)h(2n-k)$$ (1)

$$y_{low}(n)=\sum_{k=-\infty }^{\infty }x(k)l(2n-k)$$ (2)

Similarly, the following equation represents the complete convolution between the original signals and the filters with downsampling.

$$y_{high}=(x*h)↓2$$ (3)

$$y_{low}=(x*l)↓2$$ (4)

The main advantage of DWT is its capability to extract important features from the original signal, which consists of discontinuities, irregularities and sharp spikes.

### Particle swarm optimization

Particle swarm optimization (PSO) is one of the most common computational optimization techniques used to search for an optimal solution [32, 33]. The basic concept of PSO is based on nature-inspired interacting agents such as fish school and birds flock towards a certain food source. It is important to be noted that, there is no specific information related to the target location. As such, the process works randomly at the beginning and no specific assumptions are made [34]. The basic algorithm of PSO consists of a population called a swarm and candidate solutions called particles. The particles will move around the search-space based on certain mathematical formulation and their movements are guided by the best-known position in the search-space. The best known position is updated when the particle discovers a better position and the process is repeated until the optimal position is obtained.

The mathematical formulation to update the particle position is shown as follows:

$$velocity=w*velocity+r1*c1*(p\_best-x_{i})+r2*c2*(g\_best-x_{i})$$ (5)

$$w=v_{max}-[(v_{max}-v_{min}/max\_iteration]*q$$ (6)

$$x_{i}^{upd}=x_{i}+velocity$$ (7)

where

*q* = 1: max_iteration

*r*1 and *r*2 = rand(max_iteration)

*c*1 = *c*2 = 0.7

*v*_*max*_ = 0.9

*v*_*min*_ = 0.4

*p*_*best*_ = best position for each particle

*g*_*best*_ = global best position among all *p*___*best*

*x*_*i*_ = current position for each particle

$x_{i}^{upd}=update\;position\;for\;each\;particle$

The position of each particle will be observed for each iteration. If the particle comes closer to the target, the old particle (previous particle) will be replaced by a new particle (updated particle). Otherwise, the particle will remain the same.

### Artificial neural network

Artificial neural network (ANN) is an intelligent technique used for estimation, classification, prediction or forecasting. ANN is inspired based on the neural structure of the brain, which consists of millions of cells. Each of these cells is interconnected to each other and provides the ability for the brain to analyze, remember and think. In this concept, the cells are introduced as neurons, which requires an existing or pre-defined set of inputs. Each of these inputs is trained and weighted inside the neurons to be matched with the desired target [35].

**Fig 2:**
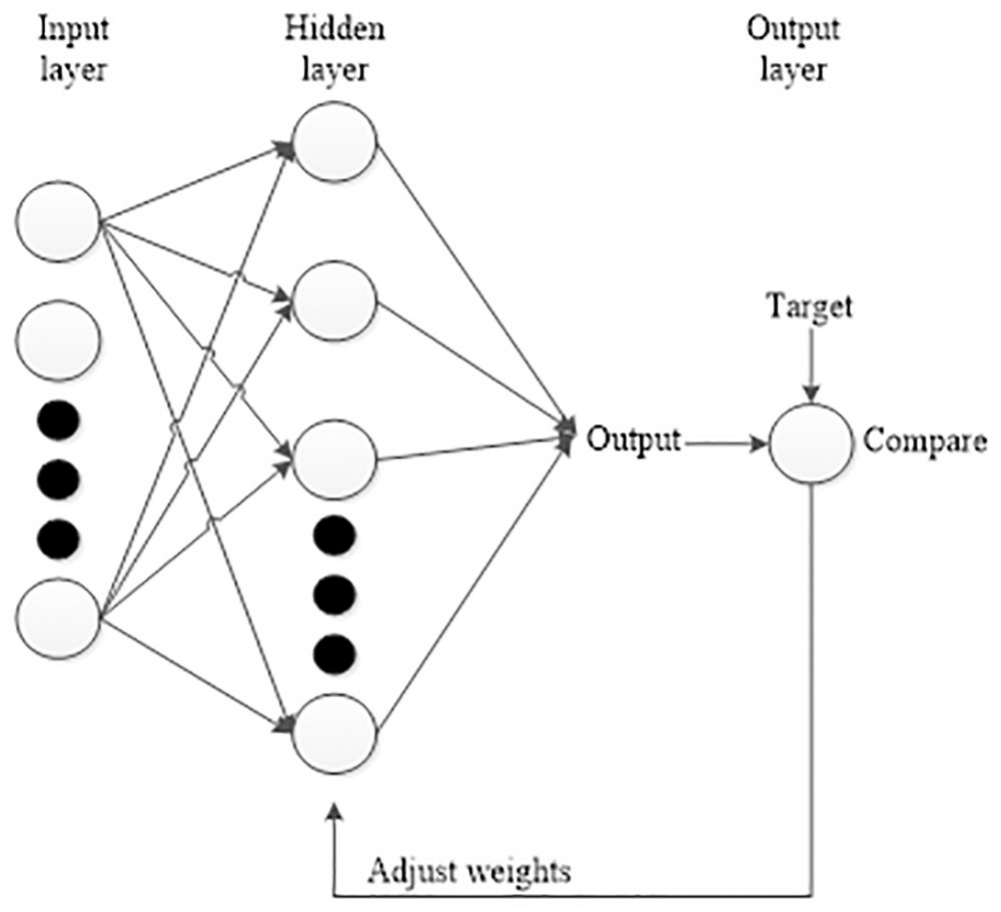
Basic artificial neural network architecture.

Fig 2 shows a typical diagram of ANN architecture which consists of three layers known as the input layer, hidden layer and output layer. The activation function used in the hidden and output layers is hyperbolic tangent sigmoid transfer function (*tansig*) and linear transfer function (*purelin*) respectively. During the training process, the input data (in the input layer) will be trained and weighted by the neurons in the hidden layer. Then, the estimated output from the training process will be compared with the desired target (in the output layer). The comparison is further evaluated based on the minimum root mean square (*rms*) error. The training process is repeated by adjusting the weights and bias inside the neurons until the estimated output and the desired target is matched with minimum *rms* error. The main advantage of ANN is the ability to implicitly learn and model the complex relationships between inputs and outputs.

## Methodology

In this paper, a hybrid approach that incorporates DWT, ANN and PSO techniques to accurately estimate the value of HIF and its distance are proposed. First, DWT is utilized to extract the detail and approximation coefficients from the three-phase voltage and current waveforms. It is noted that during the occurrence of HIF in the distribution network, the measured voltage and current waveforms have fluctuated. Further investigation shows that the fluctuation values vary with respect to the fault impedance value and the distance. As such, in this research, the fluctuated voltage and current waveforms are used as the input data to estimate the fault impedance value and its distance. The relationship between the extracted features of voltage and current waveforms is obtained by utilizing the cross-product analysis. Subsequently, the cross-product data are fed into the ANN to estimate the fault impedance and distance values. In this proposed method, the PSO technique is implemented to determine the optimal values of ANN parameters comprising the number of neurons, learning rate (*lr*) and momentum constant (*mc*).

### Feature extraction by DWT

**Fig 3:**
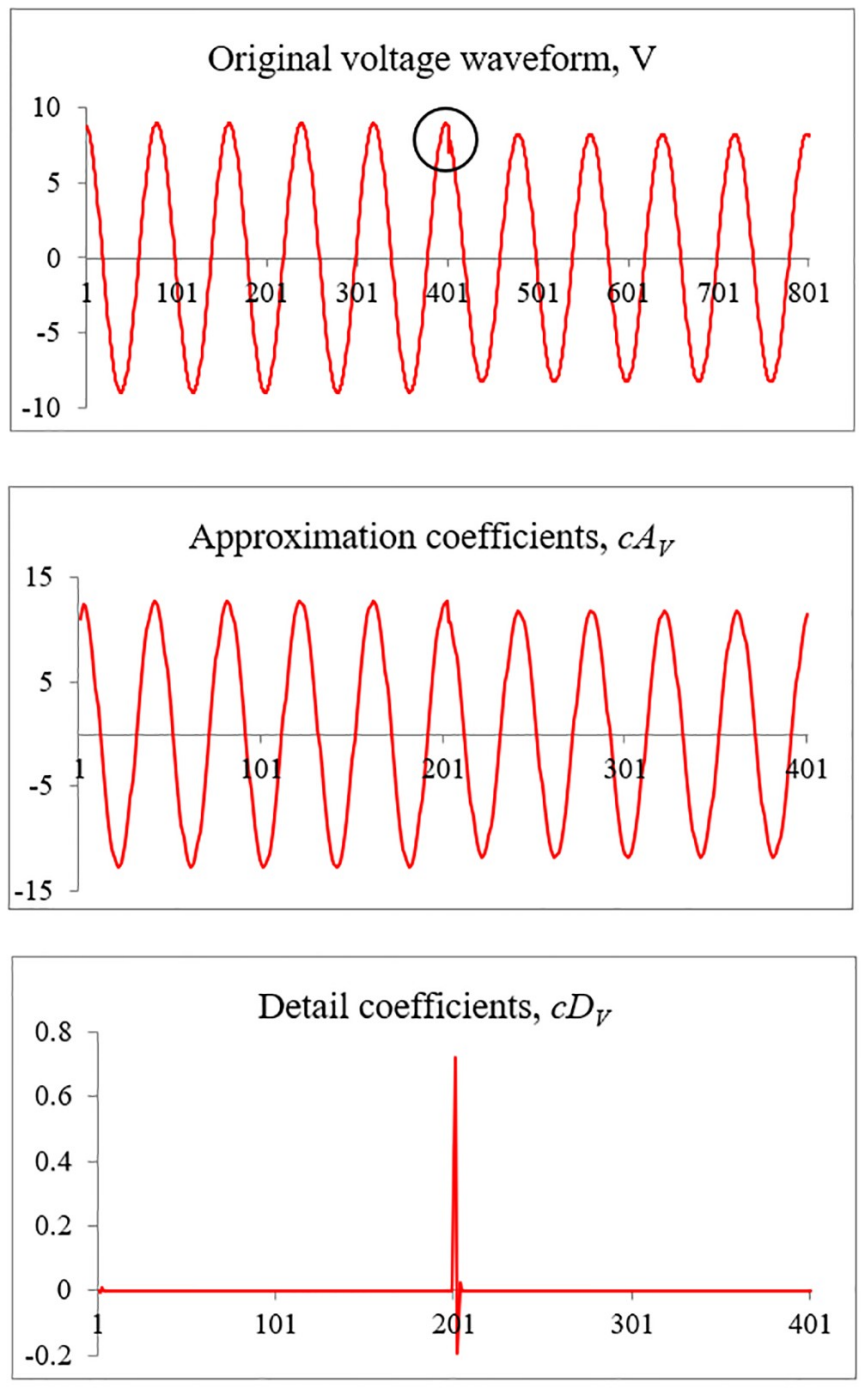
Extracted coefficients using Db4 mother wavelet. (A) Original voltage waveform. (B) Approximation coefficients. (C) Detail coefficients.

DWT is applied to extract important features from the measured three-phase voltage and current waveforms during the HIF event. The sampling rate of the waveforms is 4kHz (80 samples per one full cycle). The Daubechies fourth order (Db4) mother wavelet is selected to extract the features. Fig 3(A) shows an example of the measured phase-A voltage waveform when SLGF with fault impedance value of 50Ω is applied. The anomaly in the waveform during the HIF event is difficult to be detected by general observation. Due to this, the measured waveform is decomposed using the Db4 to detect the anomaly. The extracted features consisting of approximation and detail coefficients are shown in Fig 3(B) and 3(C) respectively. In Fig 3(C), the sharp peak indicates the starting point of an anomaly in the waveform.

**Fig 4:**
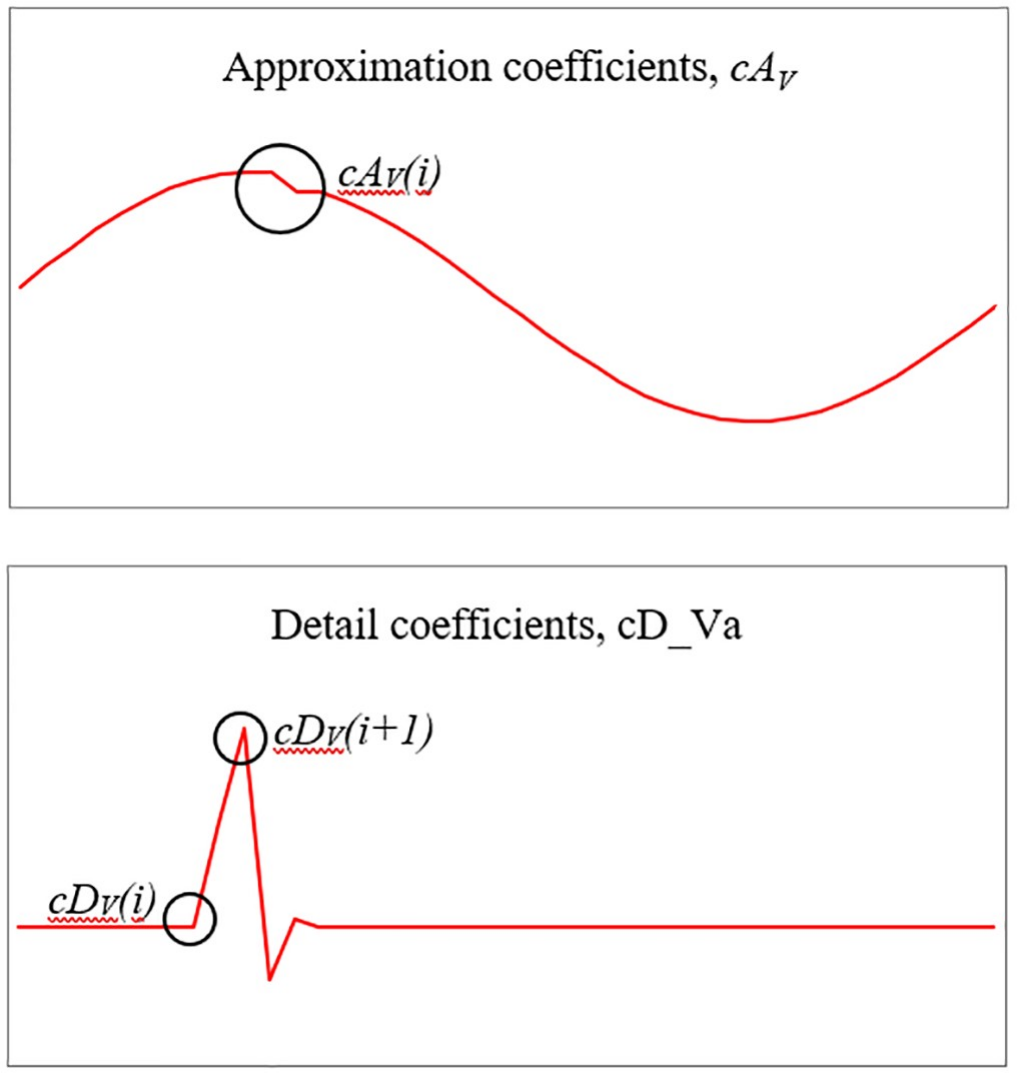
Enlarged extracted features. (A) Enlarged approximation coefficients. (B) Enlarged detail coefficients.

Fig 4(A) and 4(B) show an enlarged figure extracted from Fig 3(B) and 3(C) respectively. As shown in Fig 4(B), there is a sharp peak fluctuation in detail coefficients which can be observed when the HIF event occurs in the system. Significantly, the magnitude of the peak fluctuation varies as the fault location and impedance values vary. Thus, the variation in peak magnitude is selected as the first cue to estimate the fault location and impedance value. The first feature is the ratio of the difference between two adjacent detail coefficients (RODC), starting at the point of an anomaly as shown in Eq (8).

At the same time, a small fluctuation can be observed in the approximation coefficient as indicated by the circle in Fig 4(A). It is noted that the approximation coefficients magnitude reduces slightly after a HIF event. As such, the dip is selected as the second cue as its magnitude also changes based on the fault impedance value and location. The second feature is in the form of energy of the approximation coefficients (EAC). The energy is calculated over 80 samples of the approximation coefficients and it begins from the start of the anomaly marked by the sharp peak of the detail coefficients as shown in Eq (9). The summation of squared of 80 approximation coefficients samples (equal to one full cycle) is considered as one energy value.

The RODC and EAC are calculated as follows:

$$RODC=|\frac{cD_{{V,I}_{\left(a,b,c\right)}}(i+1)-cD_{{V,I}_{\left(a,b,c\right)}}(i)}{cD_{{V,I}_{\left(a,b,c\right)}}(i)}|$$ (8)

$$EAC=\sum_{i=1}^{80}{cA}_{{V,I}_{\left(a,b,c\right)}}^{2}(i)$$ (9)

where

${cD}_{{V,I}_{\left(a,b,c\right)}}(i+1)=first\;peak\;of\;detail\;coefficients\;of\;voltage\;and\;current\;for\;each\;phase$

${cD}_{{V,I}_{\left(a,b,c\right)}}(i)=starting\;of\;the\;first\;peak\;of\;detail\;coefficients\;of\;voltage\;and\;current\;for\;each\;phase$

${cA}_{{V,I}_{\left(a,b,c\right)}}(i)=approximation\;coefficients\;of\;voltage\;and\;current\;for\;each\;phase$

As such, there will be 12 data comprising of RODC and EAC for 3 phases of voltage and current waveforms. However, the relationship between the voltage and current is obtained through the cross-product analysis to reduce the number of data into 6. Utilizing a lower number of input data can reduce the ANN training time. The cross-product analysis is explained briefly in the next subsection.

### Cross-product analysis

Generally, the cross-product analysis which also known as vector product analysis is a binary operation that involves two vectors in three-dimensional space, normally denoted by symbol ‘x’. The resultant of cross product operation between two independent vectors is another vector that is perpendicular, known as a normal vector. In this paper, the two independent vectors are represented by voltage, V and current, I signals, whereas, the three phases (phase A, B and C) of the signal represents three-dimensional space ($\hat{\text{i}},\hat{\text{j}},\hat{\text{k}}$).

$${V=V}_{a\hat{i}}+V_{b\hat{j}}+V_{c\hat{k}}$$

$${I=I}_{a\hat{i}}+I_{b\hat{j}}+I_{c\hat{k}}$$

$$V\times I=(V_{a\hat{i}}+V_{b\hat{j}}+V_{c\hat{k}})\times (I_{a\hat{i}}+I_{b\hat{j}}+I_{c\hat{k}})$$ (10)

It can also be expressed in matrix notation.

$$V\times I=\left|\begin{array}{ccc} \hat{i} & \hat{j} & \hat{k}\\ V_{a} & V_{b} & V_{c}\\ I_{a} & I_{b} & I_{c} \end{array}\right|$$

$$V\times I=\left|\begin{array}{cc} V_{b} & V_{c}\\ I_{b} & I_{c} \end{array}\right|\hat{i}+\left|\begin{array}{cc} V_{c} & V_{a}\\ I_{c} & I_{a} \end{array}\right|\hat{j}+\left|\begin{array}{cc} V_{a} & V_{b}\\ I_{a} & I_{b} \end{array}\right|\hat{k}$$ (11)

It is observed that during the fault, both voltage and current signals are effected. Both signals have significant information related to the fault that can assist in determining the fault type, fault impedance and distance values. As such, the correlation between the voltage and current signals is obtained through the cross-product operation. It is crucial for this operation in order to reduce the number of data (RODC and EAC) without losing any significant information related to the fault as mentioned previously. Thus, a total of 6 data consisting of correlated RODC and EAC are obtained and utilized instead of 12 data. Indirectly, this can reduce ANN training time by half.

### ANN variables selection

The effectiveness of ANN is influenced by algorithm and ANN parameters such as momentum constant, learning rate and the number of neurons in hidden layers. The momentum constant, *mc* is responsible to ensure that the system is not trapped in local minima. It assists in training process acceleration by increasing the convergence speed of the system. The value of *mc* can be set in between 0 to 1 depending on the number and complexity of the data. If the value of *mc* is set too high, it will risk minima overshooting, thus making the system unstable. However, if the *mc* value is set too low, the training process will become slower.

The learning rate, *lr* is the parameter responsible to control the size of weight and bias during the training process until the estimated output matches the desired target. The proper value of *lr* can assist in accelerating the convergence of the training process. The value of *lr* can be varied between 0 to 1. If the value of *lr* is set too low, it will slow down the network learning speed. If *lr* is set too high, it will cause the weight, bias and the objective function to diverge. The value of *lr* can be adjusted based on the sum squared error (SSE) over several consequent epochs. If the value of SSE is alternating within several consequent epochs, then the value of *lr* should be decreased to slowly control the size of weight and bias. Otherwise, the value of *lr* can be increased to expedite the convergence of the training process.

The third ANN parameter which influences the ANN performance is the number of neurons in the hidden layer. It is noted that there is no limit on the number of neurons that can be used. However, if too many neurons are used in the hidden layer, it will increase the ANN training time. Besides that, it can lead to an over-fitting problem which affects the ability of ANN to generalize. Consequently, it will affect the accuracy of ANN to predict the actual output for new test data. On the other hand, if a small number of neurons is applied, it can also lead to an accuracy problem in which the system is insufficient to learn and match the input data with respect to the output data. Generally, the number of neurons is selected between the number of input data and output data. It can also be determined through trial and error approach with respect to the minimum root mean square (rms) error. Unfortunately, this approach is a time-consuming method.

There are different types of ANN learning algorithms such as Levenberg-Marquardt backpropagation, Bayesian regularization and others as mentioned in Table 1 [36]. Each of the learning algorithms has its own strength for a specific purpose such as pattern recognition or function approximation depending on the complexity of the problem.

### Determination of optimal value of ANN parameters through PSO

**Fig 5:**
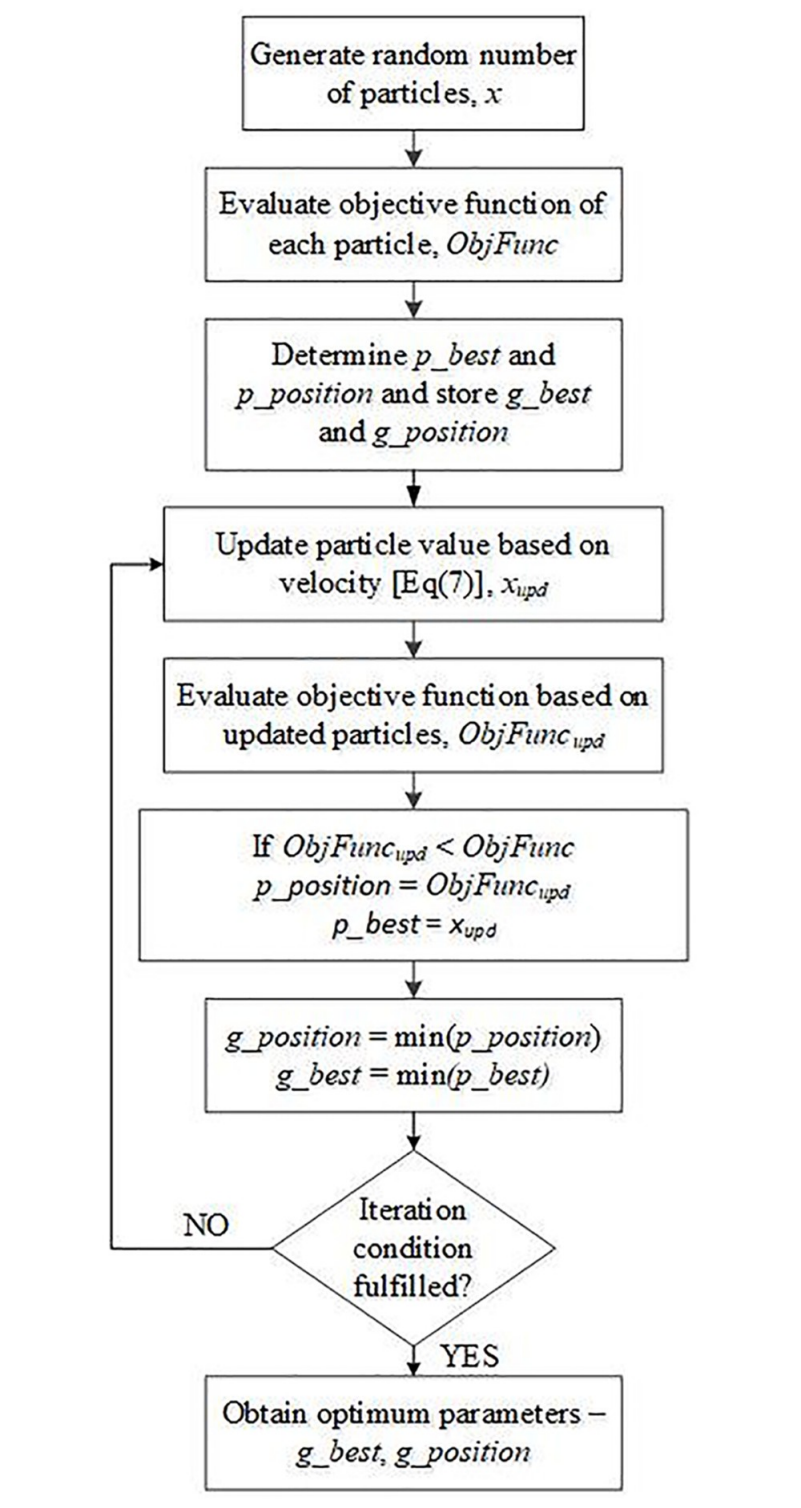
Flowchart of ANN optimization algorithm.

Fig 5 shows the flowchart of the proposed algorithm of ANN incorporating the PSO technique to determine the optimal value of ANN parameters. The proposed algorithm is initialized by generating a random number of three particles, *x* and these particles are assigned to three ANN parameters, which are *lr*, *mc* and number of neurons. The combination of these three particles is evaluated based on the objective function, *ObjFunc* as calculated using Eq (12). Through this *ObjFunc*, the local best particles, *p_best* and *p_position*, which are the ANN parameters and its *ObjFunc* respectively are determined. Among all the *p_best*, the global best particles, *g_best* and its respective *g_position* are determined and stored. The *g_best* is selected among all the *p_best* that gives the optimal value of *ObjFunc*.

The process is iterated by updating the value of the particle based on the mathematical formula given in Eq (7). The updated objective function, *ObjFunc*_*upd*_, is re-evaluated. In this step, the *ObjFunc*_*upd*_ will be compared against the previous *ObjFunc*. The *p_best* and *p_position* will be updated if the *ObjFunc*_*upd*_ is better compared to the previous *ObjFunc*. Otherwise, *p_best* and *p_position* will remain the same. Simultaneously, the *g_best* and *g_position* will be updated according to the new set of updated *p_best* and *p_position*. The process is iterated until the optimal solution is obtained or the maximum number of iterations is reached. In this study, the maximum number of iterations is set equal to 100.

The pseudocode for the proposed algorithm is given as follows:

ANN Optimization

1. Obtain random particles from PSO, *x*
2. Assign the particles to
  - learning rate, *lr*
  - momentum rate, *mr*
  - number of neurons
3. Load the training data and train the ANN.
4. Obtain the objective function, *ObjFunc*
  $$ObjFunc=\frac{{aver\_err\_fault}_{dist}+{aver\_err\_fault}_{imped}}{2}$$ (12)
  where
  $err\_{fault}_{dist}=abs(act_{{fault}_{dist}}-est_{{fault}_{dist}})$
  $err\_{fault}_{imped}=abs(act_{{fault}_{imped}}-est_{{fault}_{imped}})$
5. Update particles value, *x*_*upd*_
6. Update objective function, *ObjFunc*_*upd*_
7. Obtain the optimal solutions

## Performance of proposed method

In this section, the performance of the proposed method is investigated with two different test systems. The first and second test system consists of 18-bus and 33-bus respectively. The optimization process is carried out using MATLAB software version 8.6 and the neural network is simulated using Matlab Neural Network Toolbox^™^. In the first test system, the significance of utilizing the optimization technique is evaluated. The performance between the standard ANN and PSO-optimized ANN is observed. The effect of different types of ANN learning algorithm with the application of PSO is also investigated. Subsequently, further investigation is conducted in the second test system to show the robustness of the proposed method implemented in a bigger network.

### 18-Bus test system

**Fig 6:**
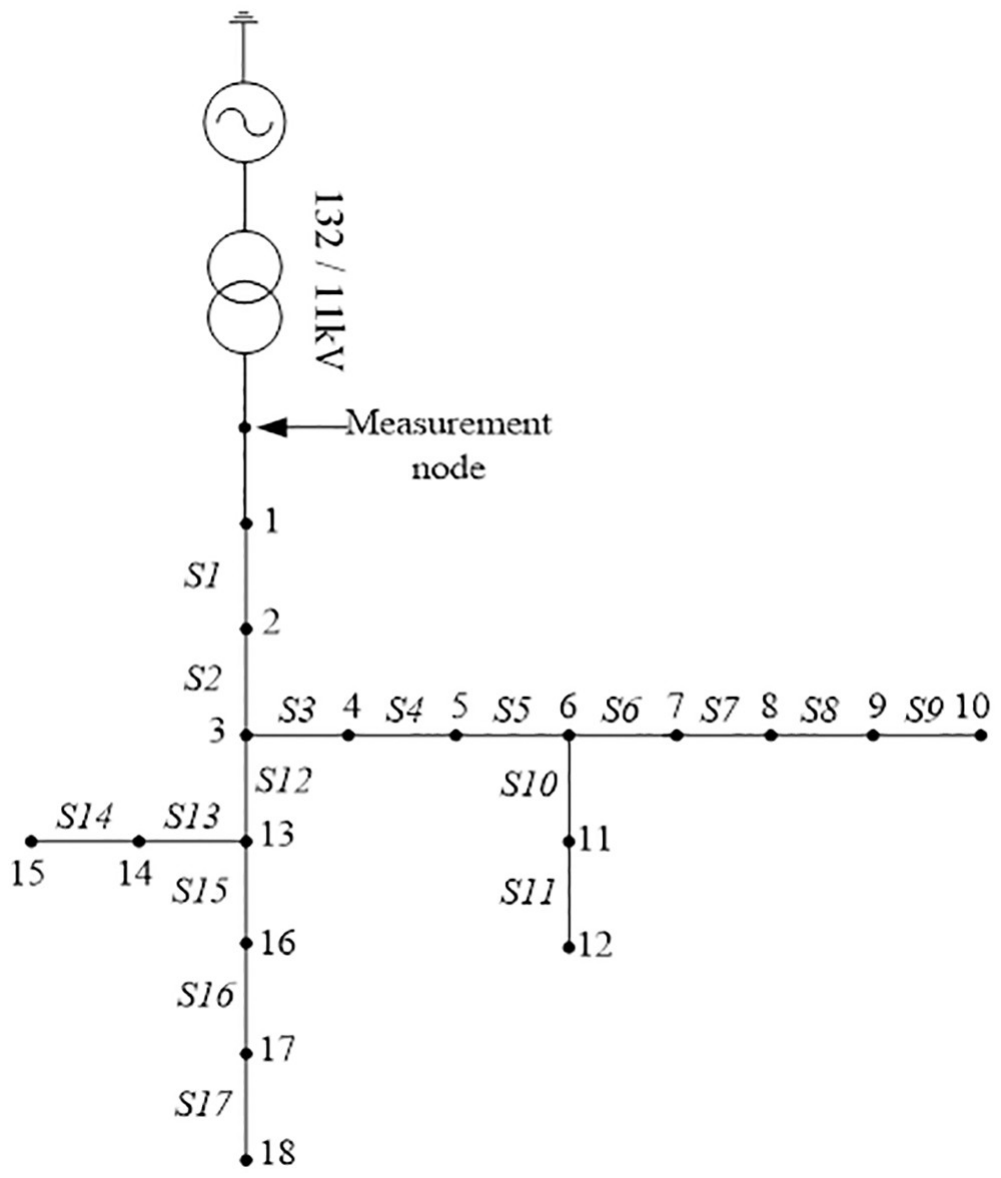
Simplified distribution network.

Fig 6 shows a simplified 132/11kV distribution network consisting of 18 buses. The network consists of 17 line sections and it is developed using the PSCAD/EMTDC software. The frequency of the system is 50Hz and the sampling frequency is 4kHz (80samples per one full cycle). Three-phase voltage and current waveforms are measured using the measurement device that can eliminate any noise. As such, in this paper, the effect of noise is not considered as it is already filtered out during the measurement process. In this investigation, different fault impedance values are applied at each node. There is a total of 187 cases consisting of 11 fault impedance values (from 50Ω to 150Ω in steps of 10Ω) and 17 nodes.

#### Analysis of the proposed method

In this study, a comparison is made between the standard ANN with PSO-optimized ANN to estimate the fault impedance and distance values. In the standard ANN, default values for *lr* and *mc* set by ANN are utilized and the number of neurons is set equal to the number of input data. In the PSO-optimized ANN, the optimal values for each ANN parameters are first determined using PSO. In both case studies, the Levenberg-Marquardt backpropagation is selected as the learning algorithm. Besides that, the same training set is used for both cases for a fair comparison. In this study, the performance for the standard ANN and PSO-optimized ANN is evaluated based on the average percentage of error as shown below:

$$Percentage\;of\;Error,PoE=\frac{\left|estimated-actual\right|}{actual}\times 100\%$$ (13)

$$Average\;Percentage\;of\;Error,APoE=\frac{\sum_{i=1}^{n}{PoE}_{i}}{n}$$ (14)

where n is the number of data.

**Fig 7:**
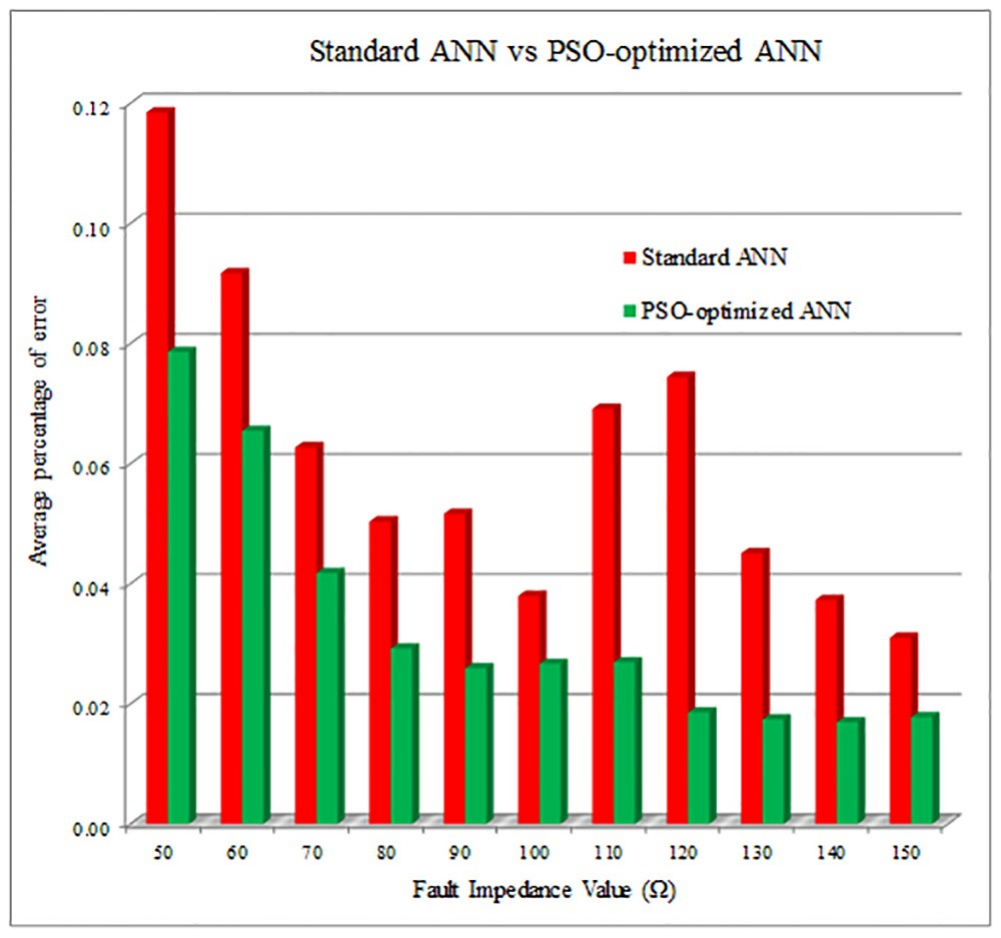
Fault impedance estimation result between standard ANN and PSO-optimized ANN.

Fig 7 shows the comparison between the standard ANN and PSO-optimized ANN results for fault impedance estimation. Based on the results, it can be observed that the *APoE* for the PSO-optimized ANN is lower compared to the standard ANN for all cases of fault impedance values. Besides that, the *APoE* for PSO-optimized ANN reduces as the fault impedance value increases.

**Fig 8:**
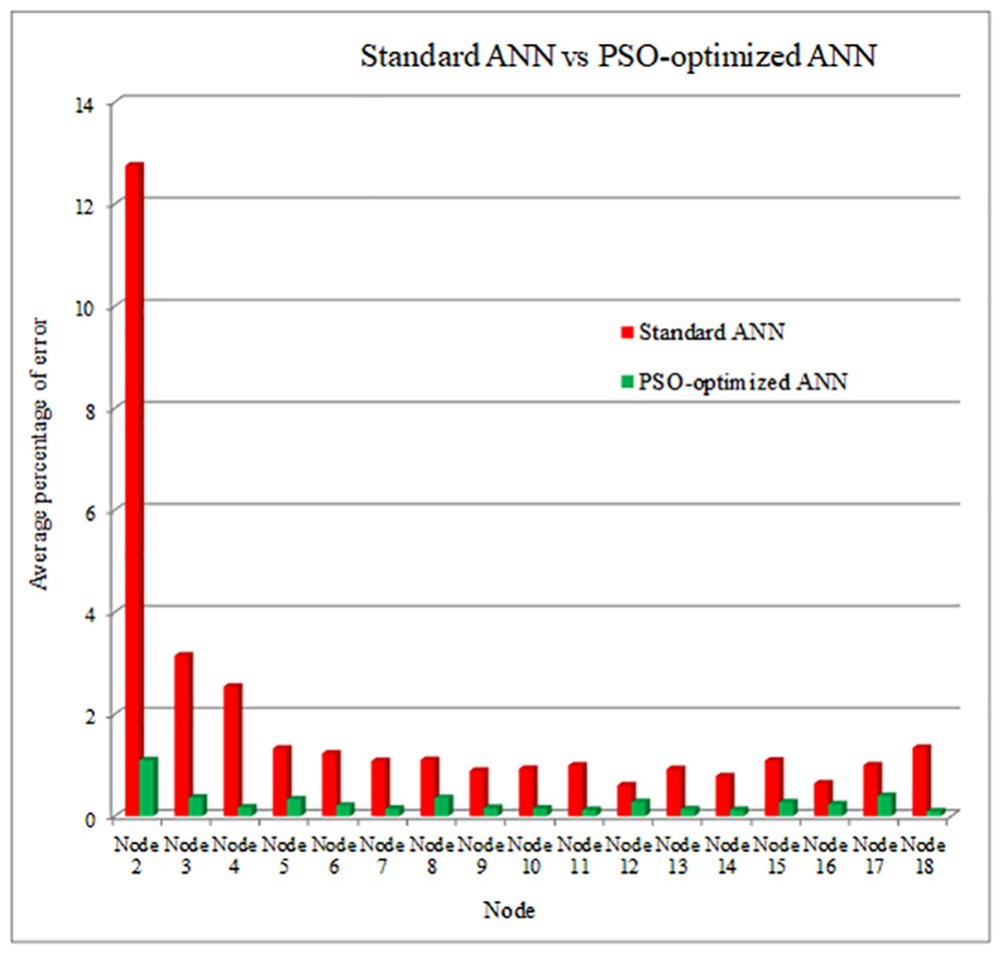
Fault distance estimation result between standard ANN and ANN-PSO.

Fig 8 shows the comparison in terms of fault distance estimation between standard ANN and PSO-optimized ANN. It can be observed that the PSO-optimized ANN delivers lower *APoE* compared to standard ANN. In general, the fault distance can be estimated accurately using PSO-optimized ANN at each node since *APoE* value is minimal (less than 2%).

The results show that the PSO-optimized ANN delivers better performance compared to standard ANN in estimating the fault impedance and distance values as depicted in Figs 7 and 8 respectively. Thus, it demonstrates the necessity of using the optimization technique to determine the optimal values of ANN parameters.

#### Different types of learning algorithm

In the previous subsection, the significance of utilizing PSO-ANN has been analyzed. In this section, the effect of different types of ANN learning algorithms such as Levenberg-Marquardt backpropagation (*trainlm)*, Bayesian regularization (*trainbr)* and others [36], are investigated as shown in Table 1. In this investigation, the HIF is applied at each node with different fault impedance values. The fault impedance and distance values are estimated by the PSO-optimized ANN utilizing different types of ANN learning algorithm. The performance for each learning algorithm is evaluated based on *ObjFunc* (Eq 12) and the results are shown in Table 1. The second to fourth column indicates the optimal values for ANN parameters tuned by PSO.

**Table 1:** *ObjFunc* based on different type of learning algorithm.

Learning algorithm [36]	*lr*	*mc*	no. of neuron	*ObjFunc*
trainbfg	0.414	0.6648	9	0.1261
trainbr	0.0065	0.6041	15	0.0394
traincgb	0.4214	0.9263	18	0.5293
traincgf	0.5272	0.4695	17	0.5041
traincgp	0.1508	0.5901	4	0.5262
trainlm	0.4706	0.2387	24	0.0174
trainoss	0.2155	0.4998	9	0.5857
trainr	0.6236	0.7638	2	0.6274
trainrp	0.7356	0.6722	14	0.5351
trainscg	0.7724	0.1303	13	0.5661

As shown in Table 1, *trainbr* and *trainlm* delivers the lowest value of *ObjFunc* with 0.0394 and 0.0174 respectively. However, further analysis is required to evaluate the consistency between *trainbr* and *trainlm* before the best learning algorithm is selected.

#### Analysis between *trainbr* versus *trainlm*

It is observed that *trainbr* and *trainlm* deliver better performance compared to the other learning algorithms. However, further investigation is required to evaluate the robustness of these two learning algorithms. The same case study as in the previous analysis is repeated 10 times for each learning algorithm to check the proposed technique’s consistency. Table 2 shows the results for both *trainbr* and *trainlm*.

**Table 2:** Robustness of *trainbr* and *trainlm* algorithms.

*trainbr*				*trainlm*			
*lr*	*mc*	no. of neuron	*ObjFunc*	*lr*	*mc*	no. of neuron	*ObjFunc*
0.0065	0.6041	15	0.0394	0.4706	0.2387	24	0.0174
0.1751	0.6973	17	0.0349	0.8064	0.5244	26	0.0156
0.812	0.3097	13	0.0374	0.5781	0.2769	24	0.0193
0.2381	0.9828	17	0.0364	0.2459	0.88	30	0.0184
0.6347	0.6566	9	0.0374	0.0078	0.8677	30	0.0166
0.323	0.5788	14	0.0355	0.6615	0.571	26	0.0187
0.6811	0.5246	22	0.0385	0.452	0	22	0.0215
0.3321	0.993	22	0.0360	0.0984	0.7597	25	0.0208
0.9513	0.8658	30	0.0390	0.5491	0.8387	25	0.0196
0.4575	0.4261	19	0.0380	0.0565	0.6745	22	0.0148

**Fig 9:**
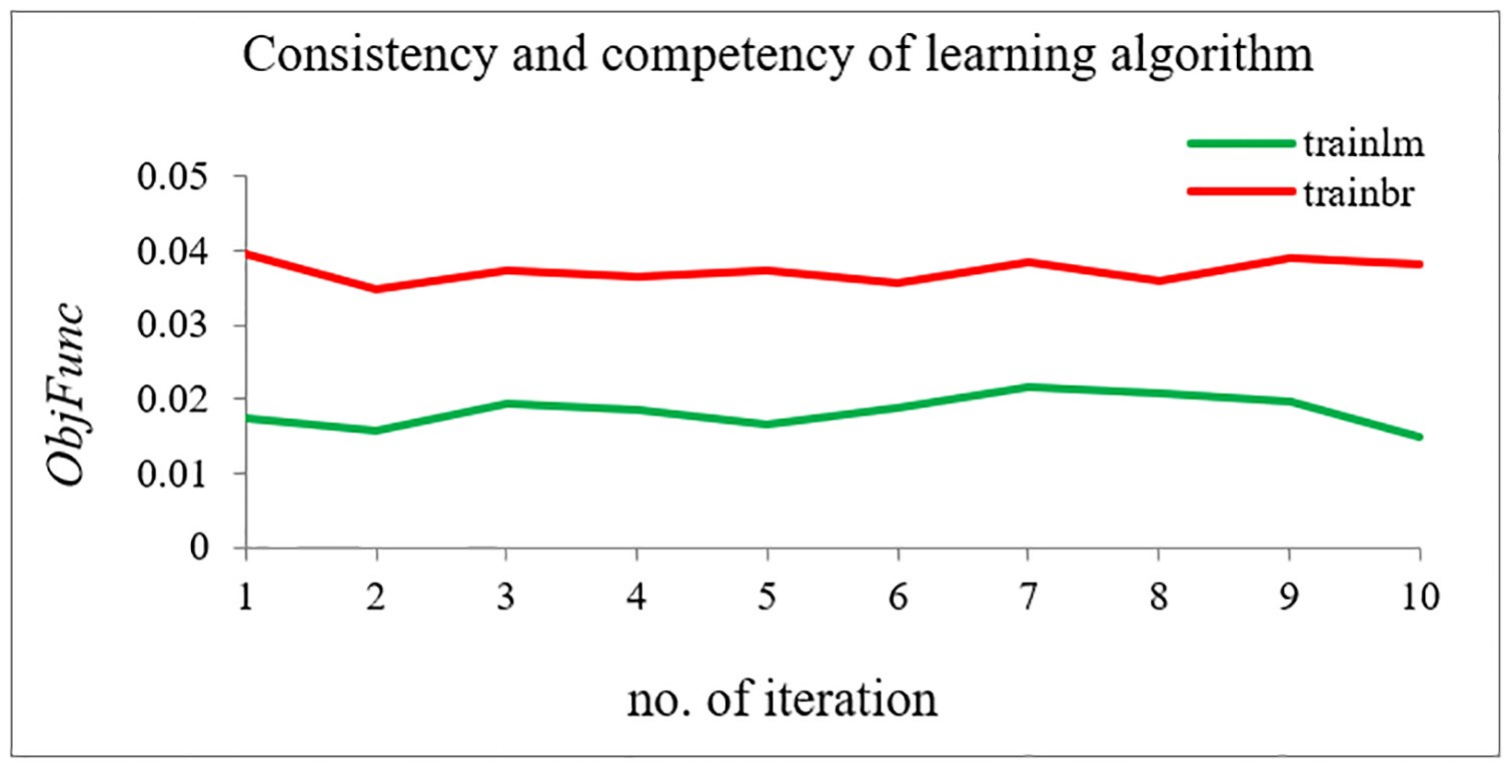
Consistency and competency between trinlm and trainbr.

Fig 9 shows the comparison between *trainbr* and *trainlm* in terms of *ObjFunc*. It can be observed that the results from both learning algorithms are consistent. However, *trainlm* is selected as the best learning algorithm as it provides the lowest value of *ObjFunc*.

#### Evaluation of the proposed method

To verify the effectiveness of the proposed method, more test cases are investigated in which the fault is applied in the middle of each line section with different fault impedance values. It must be noted that these test cases are unknown samples that are not trained by ANN. In this analysis, the performance of trained ANN to estimate the fault impedance and distance values for unknown samples are investigated. There are 170 test cases comprising of 10 different fault impedance values (55–145Ω in increment order of 10Ω) being applied in the middle of each line sections. However, only 10 out of the 170 test cases deliver poor results and these results are shown in Tables 3 and 4. It can be observed that the maximum error for fault impedance estimation is 0.333Ω as shown in Table 3. Whereas for fault distance estimation, the maximum error is 0.063km as shown in Table 4. Based on the test results, the average error of fault distance and impedance are 0.0082km and 0.0310Ω respectively. As such, the *ObjFunc* for the testing dataset is equal to 0.0196. It verifies that the proposed method can estimate the fault impedance and distance values accurately.

**Table 3:** Fault impedance estimation using PSO-optimized ANN (18-bus).

Estimated Fault Impedance (Ω)	Actual Fault Impedance (Ω)	Absolute Error (Ω)
125.333	125	0.333
145.330	145	0.330
135.307	135	0.307
115.225	115	0.225
134.838	135	0.162
124.853	125	0.147
114.862	115	0.138
144.870	145	0.130
105.107	105	0.170

**Table 4:** Fault distance estimation using PSO-optimized ANN (18-bus).

Estimated Fault Distance (km)	Actual Fault Distance (km)	Absolute Error (km)
0.313	0.25	0.063
0.307	0.25	0.057
0.201	0.25	0.049
0.298	0.25	0.048
1.081	1.125	0.044
1.084	1.125	0.041
0.290	0.25	0.040
1.090	1.125	0.035
1.094	1.125	0.031

### 33-Bus test system

**Fig 10:**
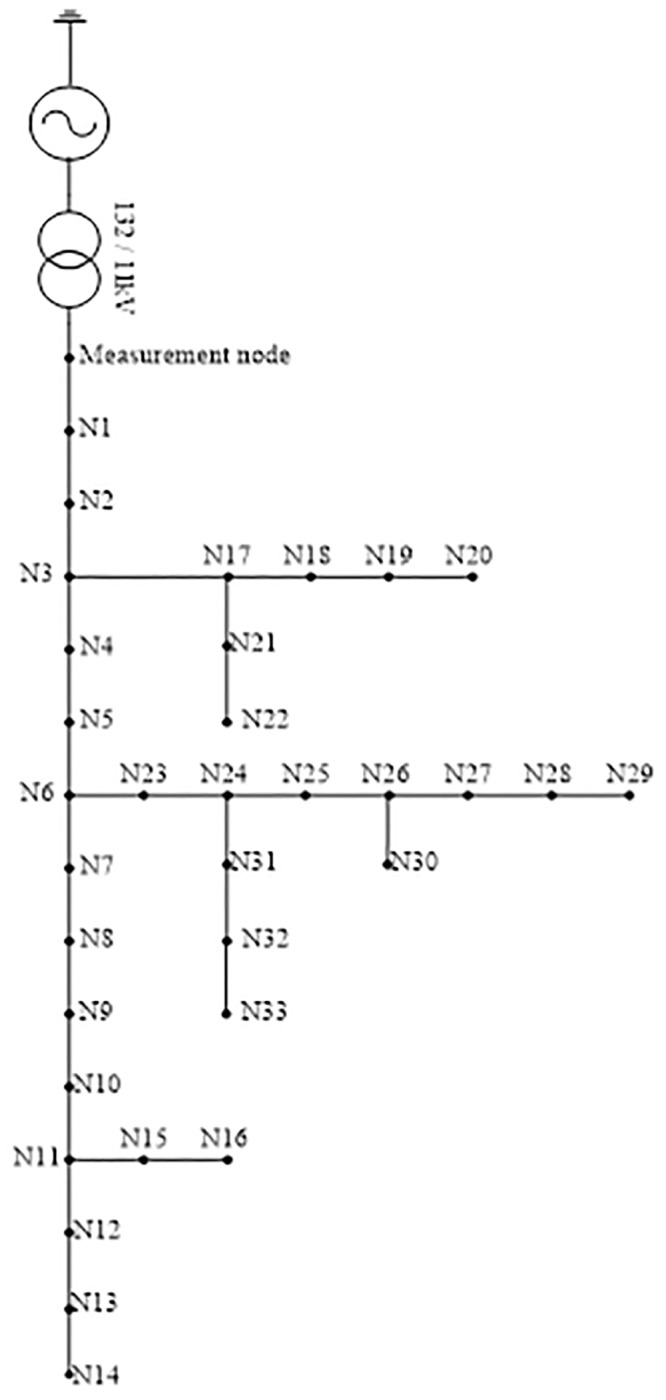
Distribution network consists of 33 buses.

In this section, the effectiveness of the proposed method is further evaluated for a larger system. Fig 10 shows a larger distribution network consisting of 33 buses. Here, 2 types of fault which are SLGF and balanced fault are investigated. These types of fault are considered since the SLGF is the most common type of fault and the balanced fault is the most severe type of fault which occurs in the distribution network [37]. SLGF is comprised of AGF, BGF and CGF, whereas for balanced fault, it comprises ABCGF and ABCF.

## Results

In this analysis, the effectiveness of the proposed method in a larger distribution network with different types of fault is evaluated. Each type of fault consists of 363 cases comprising of 11 fault impedance values (50–150Ω in increment order of 10Ω) being applied at 33 nodes. However, only the first 5 cases out of the 363 cases with the maximum value of absolute error are provided. Tables 5 to 10 shows the results for fault distance estimation whereas Tables 11 to 15 show the results for fault impedance estimation.

**Table 5:** Fault distance estimation (AGF).

Estimated fault distance (km)	Actual fault distance (km)	Absolute error (km)
***-0*.*060***	0.000	0.060
***-0*.*059***	0.000	0.059
4.001	3.945	0.056
***-0*.*056***	0.000	0.056
4.093	4.040	0.053

**Table 6:** Corrected fault distance estimation (AGF).

Estimated fault distance (km)	Actual fault distance (km)	Absolute error (km)
0.000	0.000	0.000
0.000	0.000	0.000
***4*.*001***	***3*.*945***	***0*.*056***
0.000	0.000	0.000
4.093	4.040	0.053

**Table 7:** Fault distance estimation (BGF).

Estimated fault distance (km)	Actual fault distance (km)	Absolute error (km)
***1*.*864***	***1*.*750***	***0*.*114***
1.854	1.750	0.104
1.846	1.750	0.096
1.984	1.890	0.094
1.839	1.750	0.089

**Table 8:** Fault distance estimation (CGF).

Estimated fault distance (km)	Actual fault distance (km)	Absolute error (km)
***4*.*434***	***4*.*290***	***0*.*144***
4.000	4.110	0.110
3.981	4.085	0.104
3.611	3.690	0.079
3.102	3.040	0.062

**Table 9:** Fault distance estimation (ABCGF).

Estimated fault distance (km)	Actual fault distance (km)	Absolute error (km)
***4*.*241***	***3*.*945***	***0*.*296***
4.238	3.945	0.293
4.575	4.290	0.285
4.568	4.290	0.278
4.307	4.040	0.267

**Table 10:** Fault distance estimation (ABCF).

Estimated fault distance (km)	Actual fault distance (km)	Absolute error (km)
***4*.*071***	***3*.*945***	***0*.*126***
4.070	3.945	0.125
4.158	4.040	0.118
4.407	4.290	0.117
4.155	4.040	0.115

**Table 11:** Fault impedance estimation (AGF).

Estimated fault impedance (Ω)	Actual fault impedance (Ω)	Absolute error (Ω)
***149*.*78***	***150***	***0*.*22***
110.17	110	0.17
130.16	130	0.16
120.16	120	0.16
139.84	140	0.16

**Table 12:** Fault impedance estimation (BGF).

Estimated fault impedance (Ω)	Actual fault impedance (Ω)	Absolute error (Ω)
***140*.*21***	***140***	***0*.*21***
150.20	150	0.20
149.81	150	0.19
130.19	130	0.19
140.17	140	0.17

**Table 13:** Fault impedance estimation (CGF).

Estimated fault impedance (Ω)	Actual fault impedance (Ω)	Absolute error (Ω)
***140*.*24***	***140***	***0*.*24***
150.22	150	0.22
140.21	140	0.21
110.20	110	0.20
130.20	130	0.20

**Table 14:** Fault impedance estimation (ABCGF).

Estimated fault impedance (Ω)	Actual fault impedance (Ω)	Absolute error (Ω)
***129*.*82***	***130***	***0*.*18***
129.83	130	0.17
139.84	140	0.16
130.16	130	0.16
129.85	130	0.15

**Table 15:** Fault impedance estimation (ABCF).

Estimated fault impedance (Ω)	Actual fault impedance (Ω)	Absolute error (Ω)
***140*.*20***	***140***	***0*.*20***
140.16	140	0.16
119.85	120	0.15
130.15	130	0.15
140.15	140	0.15

Table 5 shows the result for the fault distance estimation when the AGF type of fault is applied. As shown in the table, it can be observed that the maximum absolute error is 0.060km. However, the estimated fault distance is in a negative value. Since the distance cannot be in a negative value, therefore the negative estimated fault distance is changed to a zero value as shown in Table 6. As such, the new maximum absolute error becomes 0.056km.

Tables 7 to 10 show the fault distance estimation results for BGF, CGF, ABCGF and ABCF type of fault respectively. As shown in the tables, it can be observed that all the fault distance values can be estimated accurately with an error of less than 300m.

Tables 11 to 15 shows the estimated fault impedance results for different types of fault. It can be observed that the proposed method had successfully estimated the fault impedance with the maximum error of 0.24Ω, recorded during the occurrence of CGF type of fault.

### Comparison with existing methods

To justify the effectiveness of the proposed method, a comparison between the proposed method with the existing methods is conducted as shown in Table 16. The comparison is based on the maximum error of fault distance in kilometres. It is important to note that the maximum error of fault distance mentioned in Table 16 is obtained directly from the respective literature using their proposed network and technique. It can also be observed that the proposed PSO-optimized ANN delivers results with higher accuracies where the maximum error is only 0.063km for a small network whereas for a larger network, the maximum error is 0.144km and 0.296km for SLGF and balanced types of fault respectively.

**Table 16:** Comparing the proposed method to existed methods.

Technique proposed in other presented papers	Maximum error of fault distance (in km)
Wavelet, SVR [9]	0.214
Wavelet, ANN, FLS [10]	0.248
EMD, CVR [12]	0.393
Unsynchronized phasor [17]	0.095 (small network) 0.557 (large network)
Proposed method	0.063 (small network) 0.144 (large network)–SLGF 0.296 (large network)—balanced

## Conclusion

In this paper, the estimation of fault impedance values and distance based on PSO-optimized ANN is proposed. In this proposed method, important features were first extracted from the three-phase voltage and current waveforms using the DWT. The cross-product analysis is subsequently used to obtain the correlation between the voltage and current waveforms before being fed into the ANN. In this analysis, PSO was used to determine the optimal values of ANN parameters comprising of the learning rate, momentum constant and the number of neurons in a hidden layer. Performance comparison between standard ANN and PSO-optimized ANN in terms of *ObjFunc* was conducted to demonstrate the importance of utilizing the optimization technique. Furthermore, different types of ANN learning algorithms were applied to investigate their impact on the accuracy of the proposed method. Two different networks were considered to evaluate the robustness of the proposed method. Based on the simulation results, it is observed that the fault impedance and distance values can be estimated to high accuracies using PSO-optimized ANN. The maximum error is only 0.063km for a small network whereas for a larger network, the maximum error is 0.144km and 0.296km for SLGF and balanced types of fault respectively. Besides that, *trainlm* is observed to be the best ANN learning algorithm as it delivers the lowest value of *ObjFunc*.
